# Influence of Entrepreneurial Psychology and Spirit on the Cultivation of Students’ Entrepreneurial Values and Ability Under the New Media

**DOI:** 10.3389/fpsyg.2021.751045

**Published:** 2021-12-08

**Authors:** Ping Li, Xiaozhou Chen

**Affiliations:** ^1^School of Economics and Management, South China Normal University, Guangzhou, China; ^2^School of Business Administration, Guangdong University of Finance, Guangzhou, China

**Keywords:** new media, entrepreneurship, entrepreneurial values, innovation and entrepreneurship education, entrepreneurial ability

## Abstract

This paper studies the influence of entrepreneurial spirit on college students’ entrepreneurial ability and entrepreneurial values. Firstly, the impact of entrepreneurial psychology and entrepreneurial spirit on entrepreneurial values is analyzed. Secondly, the role of entrepreneurial values in Innovation and Entrepreneurship Education (IEE) is analyzed and summarized under new media. Then, based on entrepreneurial psychology and entrepreneurial spirit, a Questionnaire Survey (QS) is designed to investigate the entrepreneurial values of students in one university in Shaanxi Province, China. The QS analysis suggests that most respondents hold a positive attitude, and their attitude is on the rise. The QS results of “Reasons for entrepreneurial failure” show that 40.37, 31.9, 25.98, and 11.75% of respondents think they lack financial support, business skills, ability, and understanding of policies and laws, respectively. The QS results of “What factors influence entrepreneurial values?” reveal that 39.43% of the respondents chose the “Models of successful entrepreneurs,” successful entrepreneur models can effectively encourage students to receive IEE, followed by 28.94% who choose “Achieve their own life goals.”. In terms of “Solutions against entrepreneurial risks,” nearly 70% of the students have chosen the negative options. In terms of “Which is the most important entrepreneurial quality?”, more students choose entrepreneurial values rather than entrepreneurial quality, proving that students generally lack entrepreneurial values. Given these problems, corresponding countermeasures are put forward to strengthen entrepreneurial psychology and IEE, in an attempt to cultivate college students’ entrepreneurial values and entrepreneurial ability under the new media. The results provide some data support for the impact of college students’ entrepreneurial values and entrepreneurial ability.

## Introduction

Entrepreneurship is the source of social development. Different regions take distinct methods to carry out Innovation and Entrepreneurship Education (IEE). In the west, IEE involves students’ value training, quality education, and ideological education. Meanwhile, Westen schools also design clear educational objectives and form a unique and complete structural system to cultivate students’ entrepreneurial values and entrepreneurial ability. However, there are still some gaps in the training system of students’ entrepreneurial values and entrepreneurial ability between China and the West. Therefore, Chinese educators and researchers might need to absorb international education experience and learn from foreign education systems to improve students’ self-learning levels. In the context of new media, many researchers, including Chinese and Western scholars, have explored students’ entrepreneurial values in entrepreneurial practice. Wang and Zhang (2020) believed that personalized education was an innovative education method that respected individual differences. With the progress of the times, the number of innovative and entrepreneurial talents had become one of the key indicators of comprehensive national strength. Therefore, while cultivating students’ ability to think and solve problems independently, there was a need for higher institutions to respect the creativity and subjectivity of college students to lay a solid foundation for students’ future innovation and entrepreneurship. Additionally, some scholars have compared and analyzed the current situation, existing problems, and solutions of personalized education between China and the West to encourage students’ autonomy. Jin et al. (2019) proposed a new model, which tested the effect of IEE on 308 students in a Chinese university from three dimensions of entrepreneurial ability, entrepreneurial barriers, and entrepreneurial intention. As a result, the effectiveness of IEE had been verified in improving entrepreneurial ability, reducing entrepreneurial barriers, and changing entrepreneurial intention. Wu et al. (2018) argued that IEE could help rural enterprises create higher economic value. The survey found that the respondents believed that IEE had an impact on the local regional entrepreneurship ecosystem. Most respondents benefited from the course, but the degree of benefit decreased as the IEE course came to a closure. Hagebakken et al. (2021) contended that IEE was the key to stimulating people’s interest in entrepreneurship. He analyzed the characteristics of entrepreneurs, found a customized training mode for rural young entrepreneurs suitable and agricultural industries, and specifically analyzed the factors affecting the entrepreneurship of agricultural college students. The results showed that risk-taking, organizational ability, leadership, and efforts affected their determination to continue to develop agriculture, but opportunities, patience, and other factors had little effect. Ayu and Nauly (2020) found that teachers and students had significant differences in all dimensions of the employability scale and entrepreneurial ability scale, but there was no significant difference in gender, which indicated that there was a close relationship between college students’ entrepreneurial ability and employment.

This paper is divided into four sections. The first section is the introduction; starting from the background of new media, it introduces in detail the research status and significance of students’ entrepreneurial values and entrepreneurial ability training from domestic and international perspectives and outlines the organizational structure. The second section introduces the research methods, puts forward the IEE theory, explains the characteristics and current situation of new media technology, and designs a Questionnaire Survey (QS) to test students’ entrepreneurial values. The third section discusses the research results and analyzes college students’ entrepreneurial values from different angles through QS. The fourth section draws conclusions, including actual contributions, limitations, and prospects.

## Content and Significance of Innovation and Entrepreneurship Education, and Entrepreneurial Values

### Innovation and Entrepreneurship Education and Entrepreneurial Values

With rapid economic development, people’s understanding of entrepreneurship has been in constant variations. Today, entrepreneurship specifically refers to the establishment of new enterprises, a process of new career exploration. In essence, entrepreneurship is an innovative process, and entrepreneurs tend to have a positive outlook on life (Bogatyreva et al., 2019; Whitcher, 2019) following decades of development, entrepreneurship is still a new research field with fascinating prospects, but there has not been a consensus definition, and researchers generally hold that entrepreneurship contains three elements: product development, innovation, and entrepreneurial spirit. Firstly, entrepreneurship can promote employment, and create more jobs, promoting the sustainable and steady development of the social economy (Karayol and Taze, 2020). In turn, successful entrepreneurship also needs various forms of social assistance. Evaluated students’ experience of using mobile-based Classroom Response System (CRS) technology in entrepreneurship courses (Wu W. et al., 2019). Secondly, in terms of innovation, entrepreneurship itself is an innovative process. Under the booming information age, successful entrepreneurs are no doubt innovators as well. Lastly, entrepreneurial spirit is the determination to tackle difficulties and risks of entrepreneurs during the entrepreneurial process, which is the foundation of entrepreneurship (Rogoza et al., 2018; Gonzalez, 2019).

Entrepreneurship can also be decomposed from the dimension of values: including the entrepreneur value, national value, and social value, the realization of which is the primary driving force for most college entrepreneurs (Prihatini and Hidayatullah, 2019; Eklund et al., 2020). Researchers interpret entrepreneurial values from different perspectives. Some point out that entrepreneurial values are entrepreneurs’ recognition of entrepreneurial goals based on their individual needs and the criteria for entrepreneurial behaviors. Accordingly, college students’ entrepreneurial activities originate from their entrepreneurial needs, like survival and starting a business (Morgan, 2019; Wang, 2019). In particular, the sense of social responsibility is essential for successful entrepreneurship apart from strong individual demand. Correct values are arguably the foundation of entrepreneurship and the key competitive advantage of entrepreneurs, guiding the entrepreneurial process. Explored the use and user satisfaction of social media in entrepreneurship from the perspective of learners (Wu and Song, 2019). Thereupon, the main content of college entrepreneurship in China is constructed from the following aspects:

(1) Indigenous emotion. From ancient times to today, people always have been endowed with different missions and emotions of that era, and indigenous emotion, in particular, has deeply affected the Chinese, which is, thus, part of Chinese college students’ entrepreneurship that serves the Chinese dream of rejuvenation. Surely, the entrepreneurial path is paved with challenges and risks, as well as temptations from mammonism, hedonism, and egoism, with which the entrepreneurial process might come to a premature end unless college entrepreneurs have a strong indigenous emotion and willpower. Besides, Chinese college entrepreneurs are supposed to have the comprehensive ability of national cultural integration. Entrepreneurship bolsters individuals’ sense of self-worth, and it is deemed an act to serve the Chinese nation (Lee and Raschke, 2020; Yang, 2020).
(2) Social dedication. Social dedication is the essential feature of socialist professional ethics and the major content of entrepreneurial values in China. It has been argued that the value of life first lies in social dedication, and as a social unit, people should not merely strive for their own happiness but also endeavor to contribute to national well-being. With the upcoming new era, more social spheres are supporting entrepreneurial activities, in which college students are expected to play a leading role. Therefore, college IEE should help the students acquire the skills of innovation and entrepreneurship under the new media background and develop a sense of social responsibility (Wang, 2020b).
(3) Innovation and development. Innovation is the soul and source of national development, which should be integrated into contemporary college students’ entrepreneurial process. Meanwhile, innovation focuses on newness, improvement, and spread of ideas or technologies, and the purpose is to create business value, namely, the incremental improvement to existing products. China’s future development is believed to lie in the hand of college students who possess comprehensive qualities, especially, innovative spirit. Therefore, the education of entrepreneurial values in higher institutions under the new media should pay more attention to students’ entrepreneurial development, strengthen their independent innovation ability, and stimulate their motivation for innovation and entrepreneurship (Wu, 2020).
(4) Risk-taking spirit. Entrepreneurship is a long-term enduring process during which entrepreneurs face various challenges (risks) and should keep on working to transform their environment. Fortunately, the risk-taking spirit can be cultivated through acquired efforts, which, therefore, should be strengthened through college education of innovation and entrepreneurship under the background of new media (Klein, 2019).
(5) Collectivism. In today’s ultra-competitive society, challenges and opportunities go hand in hand. Cross-border cooperation is becoming increasingly important in economic globalization, and the sense of cooperation and collaboration is growing into the basic requirements for entrepreneurial talents. Therefore, during the education for students’ innovation and entrepreneurship, higher institutions should pay full attention to cultivating students’ entrepreneurial awareness and competitiveness, as well as their cooperation ability (Qian et al., 2018).

Further, entrepreneurial psychology also has a particular impact on the formation of college students’ entrepreneurial values. It has been argued that the psychological quality of entrepreneurial talents should be divided into seven dimensions: confidence, willpower, imagination, exploration, flexibility, and cooperation awareness. Others believe that entrepreneurial psychology should include innovative thinking, basic theoretical knowledge, innovative will, and innovative ability. Entrepreneurial psychology during entrepreneurship involves knowledge, thinking, ability, concept, and will. The cultivation of college students’ entrepreneurial psychology can be divided into four parts: entrepreneurial awareness, entrepreneurial will, entrepreneurial ability, and entrepreneur personality. The influence of entrepreneurial psychology on college students’ entrepreneurial values can be summarized into seven aspects: (1) innovation, entrepreneurship, and cognition, this aspect includes innovative thinking, cognition of potential innovation and entrepreneurship opportunities, entrepreneurs’ cognitive deviation, and relevant thinking training; (2) innovation, entrepreneurship, and personality characteristics, this aspect includes the entrepreneurs’ personalities in different stages of the entrepreneurial process, the prediction of personalities, and the entrepreneurial failure-prone personalities; (3) innovation, entrepreneurship, and ability, this includes the personal ability required for innovation and entrepreneurship, the training mode of personal ability, and the role of unique ability in a specific period; (4) innovation, entrepreneurship, and psychopathology, this aspect includes the relationship between entrepreneurship and psychopathology, the prevalence of entrepreneurs’ psychological disorders, entrepreneurs’ psychological regulation, common psychological disorders, and entrepreneurs’ perception of employees’ psychological state; (5) innovation, entrepreneurship, and emotion, this aspect includes the impact of emotion on cognition, the test and cultivation of EQ; (6) innovation, entrepreneurship, and leadership psychology, this aspect includes the research on the characteristics of leaders, the matching degree between leaders and the environment, the quality of leaders, and the leadership of college students; (7) innovation, entrepreneurship, and positive psychology, this aspect includes the content research of positive psychology, the impact of positive psychology on entrepreneurs, and the intervention and training research of positive psychology.

### Characteristics and Status of the New Media Technology

With the advancement of computer and information technologies, various types of new media forms emerge, which are attracting more audiences through interactive changes to the traditional techniques and communication modes over the Internet. Used a quasi-experimental design and qualitative methods to test the effectiveness of the PowToon tool to present business plans. New media technology has three technical characteristics: (1) sharing, that is, the new media technology breaks the time-spatial limitation by providing people with abundant information exchange. (2) Diversity, that is, new media forms are more diverse, which can be flexibly expressed through a single form or combination of audio, video, and image, among others (Ding, 2020). (3) Multi-directional interaction, that is, the information sending terminal always changes from a single point to multi-point and multi-direction (Wu Y. et al., 2019; Xiao et al., 2019).

College students are considered to be a well-educated and more knowledgeable social group with a strong desire and competency to explore new things and ideas, who are very active in the use of new media. With the development of the Internet, the new media, especially, mobile social media, has gradually become the main information acquisition and exchange channel for college students. New media have a great impact on the interpersonal communication, learning and life, and innovative thinking of college students (European Physical and Rehabilitation Medicine Bodies Alliance, 2018; Soegoto and Luckyardi, 2020). Thus, there is a need for higher institutions to carry out innovation and entrepreneurship activities through the new media to help students improve their innovation and entrepreneurship ability. Nowadays, college IEE requires more information sharing and multiple interactions. New media has brought various new resources along with challenges for college IEE (Huang Q., 2019). For example, higher institutions can implement the online platform of IEE based on new media technology, such as Massive Open Online Courses (MOOCs), which puts classroom teaching over the Internet platform and changes traditional face-to-face education into online education. The IEE platform can provide information technology services for students through the Internet of Things (IoT), cloud computing technology, encryption algorithm, and other technologies, and integrate educational resources and scientific research, together with the latest entrepreneurship policies and theories to create targeted education courses for students (Huang J., 2019). New media technology has great application potential in college IEE because it can integrate massive teaching content and classic cases to provide extended after-class readings for the benefit of students. Moreover, with super interactivity, the new media can provide rich entrepreneurial information for college students, as well as various entrepreneurial channels to encourage college students’ entrepreneurship (Yan, 2018).

### Questionnaire Survey Design of College Students’ Entrepreneurial Values

The research subject is the college students from one university in Xi’an. Totally, 150 QSs are distributed, and 142 valid QSs are recovered, with an effective rate of 94.6%, of which the Science and Engineering students account for 52.3% of the total sample, the Economics and Management students account for 29.1%, and other majors account for 18.6%. The QS is composed of four parts: the first part is the basic information of the respondents, including gender, grade, school, major, and family location; the purpose is to test whether there are significant differences in observed variables in different populations. The second part is to master the students’ cognition of entrepreneurship. The third part is the College Students’ Innovative Spirit Scale, which is used to measure the innovative spirit of the respondents. The fourth part is College Students’ Entrepreneurial Ability Scale, which is used to observe students’ entrepreneurial ability. College Students’ Innovative Spirit Scale includes four dimensions: flexibility (questions 1, 4, 6, 12, 15, 16, and 22), standard innovation (questions 2, 8, 17, 19, 21, 23, and 25), criticism (questions 3, 7, 11, 13, 18, and 20), and reflection (questions 5, 9, 10, 14, and 24), with a total of 25 questions. For fear of thinking set that might affect the measurement results, questions 4, 11, 13, 14, 20, 22, and 25 adopt the reverse scoring method. The Likert’s five-point scoring method is adopted, in which “completely inconsistent,” “not quite consistent,” “uncertain,” “relatively consistent,’ and “fully consistent” are assigned “1–5” points, respectively. The average total score of the scale is used to measure the level of innovation spirit: 1–2 points indicate low level, 2–3 points represent middle and lower level, 3–4 points refer to the middle and upper level, and 4–5 points stand for high level. College Students’ Entrepreneurial Ability Scale includes opportunity exploration ability (questions 1, 2, 3, and 4), organization and management ability (questions 5, 6, and 7), strategic decision-making ability (questions 8, 9, and 10), resource integration ability (questions 11, 12, 13, and 14), innovation and creativity ability (15, 16, and 17), and frustration tolerance ability (questions19, 20, 21, and 22), totaling 22 questions. Likewise, Likert’s five-point scoring method is employed: “completely inconsistent,” “not quite consistent,” “uncertain,” “relatively consistent,” and “fully consistent” are assigned 1–5 points, respectively; the average total score of the scale is used to measure entrepreneurial ability: 1–2 points indicate low level, 2–3 points refer to the middle and lower level, 3–4 points mean middle and upper level, and 4–5 points stand for high level. Further, SPSS 25.0 is used to statistically analyze the sample data.

Main contents of the QS:

(1) Factors influencing college students’ entrepreneurial choice: (A) realization of personal ideals and values; (B) high degree of freedom; (C) high earrings; (D) poor employment situations and affected by others.
(2) Essential elements in the entrepreneurial process: (A) education; (B) ability; (C) relationship; (D) others.
(3) Solutions against entrepreneurial risks: (A) take a positive attitude; (B) hesitate; (C) give up; (D) let nature take its course.
(4) What factors lead to entrepreneurial failure? (A) lack of financial support; (B) lack of basic business skills; (C) lack of ability (D) lack of understanding of policies and regulations.
(5) What factors influence entrepreneurial values? (A) achieve their own life goals; (B) models of successful entrepreneurs; (C) government influence; (D) school influence.
(6) Which is the most important entrepreneurial quality? (A) innovation-driven development; (B) adventure; (C) win–win cooperation and perseverance; (D) honesty and trustworthiness.
(7) What types of new media do college students use? (A) Sina portals; (B) QQ; (C) Wechat; (D) others.
(8) Why do college students use new media? (A) entertainment; (B) subscribe to the current news; (C) propagation; (D) others.
(9) Time distribution of college students’ use of new media: (A) 1 ∼ 2 h; (B) 3 ∼ 4 h; (C) 5 ∼ 6 h; (D) More than 6 h.

## Analysis and Optimization of College Students’ Entrepreneurial Values

### Reliability and Validity Analysis

Table 1 shows the statistics of the reliability of the scale.

**TABLE 1:** Reliability statistics.

Cronbach’s alpha	Standardized Cronbach’s alpha	Number of items
0.791	0.761	7

Table 1 demonstrates that the Cronbach’s Alpha coefficient is used to verify the internal consistency and reliability of the QS, which ensures the scoring consistency of each item. The measured value of α is 0.797, indicating that the QS has good reliability and internal consistency.

### Analysis of the Results of the Questionnaire Survey on Entrepreneurial Factors

**FIGURE 1:**
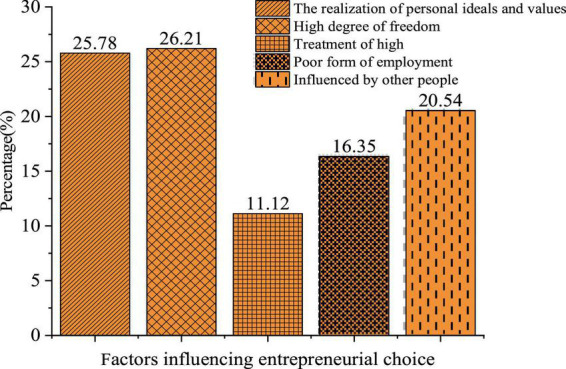
Factors influencing the entrepreneurial choice.

**FIGURE 2:**
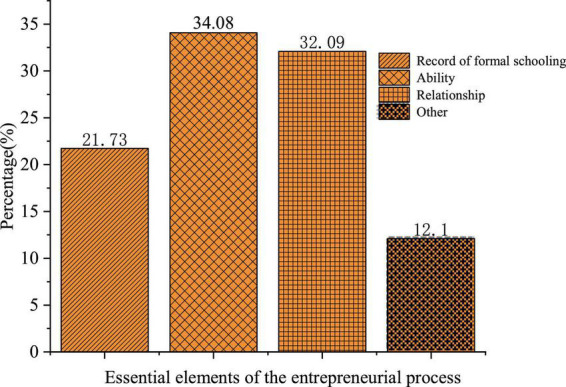
Essential elements for the entrepreneurial process.

**FIGURE 3:**
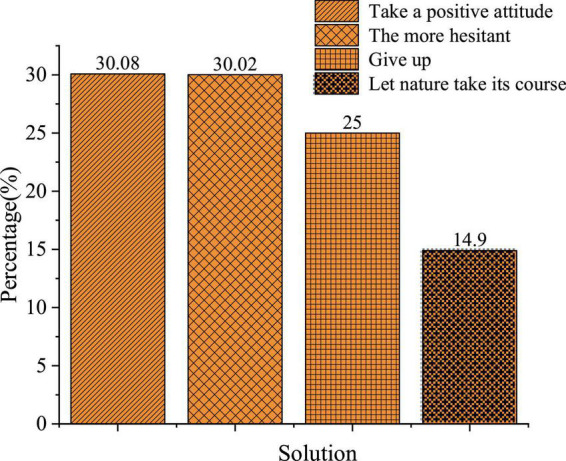
Solutions against entrepreneurial risks.

The QS results of the entrepreneurial factors are shown in Figures 1–3.

Figure 1 presents the distribution of students’ answers to factors affecting entrepreneurial choice. 25.78% of them choose “The realization of personal ideals and values,” which is also the values of the current IEE; 26.21 and 11.12% of them choose “High degree of freedom” and “High earnings,” respectively, with some utilitarian ideas; while 16.35% of them choose “The poor employment situations,” under which self-employment is believed to a new choice. Overall, college students’ entrepreneurial values are positive, and “The realization of personal ideals and values” is most influential to their entrepreneurial choices, while, by comparison, they pay lesser attention to the realization of their national and social values.

Figure 2 illustrates the distribution of students’ answers to the essential elements of the entrepreneurial process. Specifically, 21.65, 33.94, and 31.97% of them think education, ability, and relationship are the most essential, respectively. Hence, most college students believe that education and ability are more important in the entrepreneurial process, but other factors, like money, are also indispensable. It has been argued that an entrepreneur’s ability and education are internal factors while other factors are the external factors, which are both essential for successful entrepreneurship.

Figure 3 exhibits the distribution of students’ answers to the solutions against entrepreneurial risks. 30.35 30.29, 25.23, and 14.13% of them choose “Take a positive attitude,” “Hesitant,” “Give up,” and “Let nature take its course,” respectively. Thus, college students lack social experience and their risk-bearing ability is incompetent; nearly 70% of the respondents have shown no positive attitude against entrepreneurial risks. Given this, there is a need for higher institutions to attach great importance to the guidance of values and entrepreneurial psychology in IEE.

### Entrepreneurial Values Questionnaire Survey Results Analysis

**FIGURE 4:**
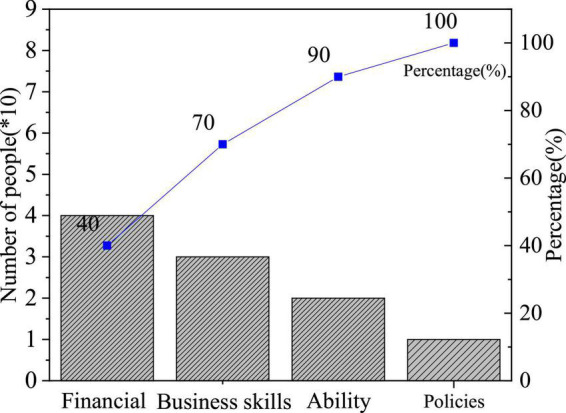
Factors of entrepreneurial failure.

**FIGURE 5:**
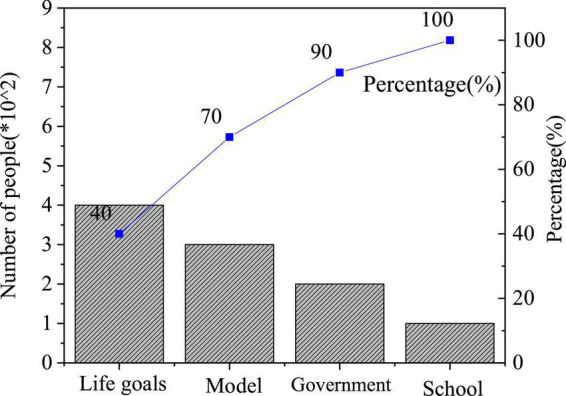
Factors influencing entrepreneurial values.

**FIGURE 6:**
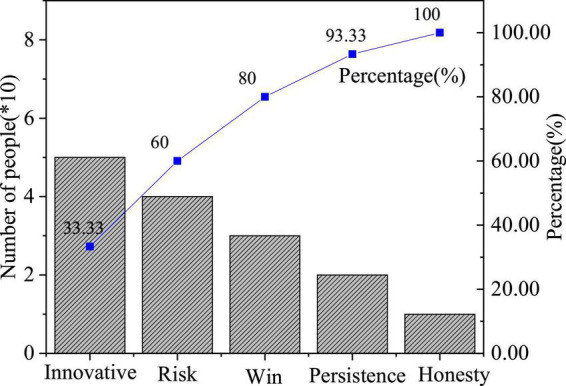
Importance of entrepreneurial quality.

The QS results of entrepreneurial values are shown in Figures 4–6.

Figure 4 shows the distribution of students’ answers to “What factors lead to entrepreneurial failure?”. Specifically, 40.37, 31.9, 25.98, and 11.75% of them think they lack financial support, business skills, ability, or understanding of policies and laws, respectively. Thus, entrepreneurship is a practical process, and IEE needs to be combined with practice; also, it has been recognized by students that the skills and ability of entrepreneurs play an important role in the entrepreneurial process; besides, financial support affects the whole life cycle of entrepreneurship. Overall, most students hold correct entrepreneurial values.

Figure 5 displays the distribution of students’ answers to “What factors influence the entrepreneurial values?”: 39.43% of them choose “Models of successful entrepreneurs,” showing that the power of the model is infinite, and entrepreneurial spirit can strongly promote entrepreneurship, followed by 28.94% who choose “Achieve their own life goals”; those who have chosen “Government influence” or “School influence” both account for over 15%. Thus, as the enforcement authorities of national rights, the government supports college students’ entrepreneurship and affects the development of IEE by implementing relevant policies; higher institutions might be exactly where students form correct entrepreneurial values.

Figure 6 reveals the distribution of students’ answers to “Which is the most important entrepreneurial quality?”. Overall, students who choose entrepreneurial values outnumber those who choose practical entrepreneurial ability. Hence, students generally lack entrepreneurial values, and there is a need for higher institutions to pay attention to the cultivation of students’ spiritual quality in the education of entrepreneurial values. The value and quality of entrepreneurs are of great significance to the development of the country and individuals.

### The Questionnaire Survey Results of College Students’ Use of New Media

**FIGURE 7:**
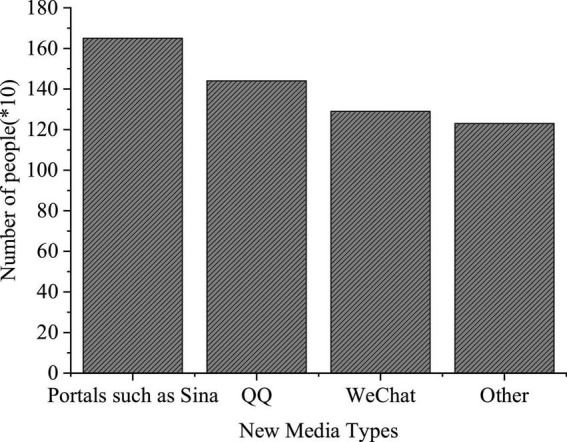
Questionnaire Survey (QS) of the types of new media used.

**FIGURE 8:**
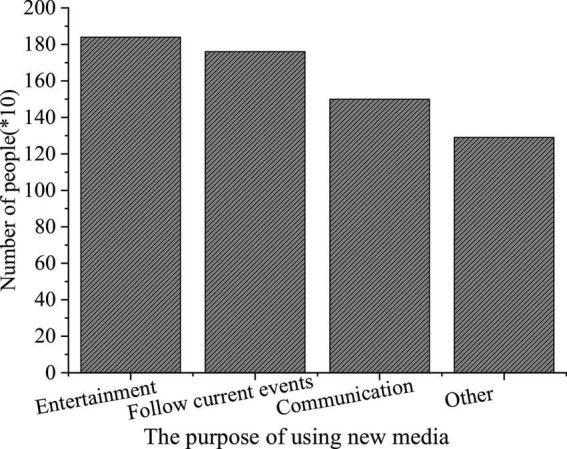
Questionnaire Survey of purposes for using new media.

**FIGURE 9:**
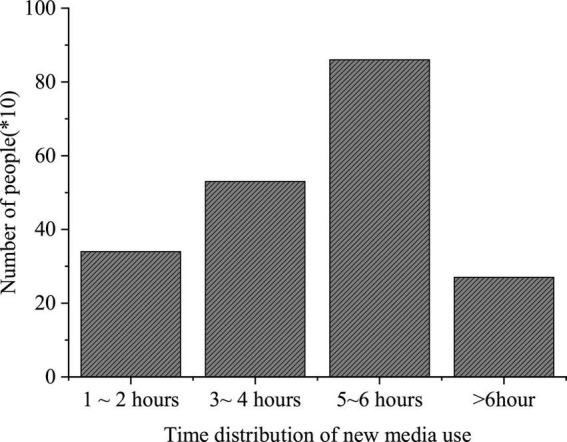
Questionnaire Survey of the new media utilization time.

The QS results of college students’ use of new media are shown in Figures 7–9.

Figures 7–9 imply that such new media platforms as Sina portal, QQ, and WeChat are widely used among college students, with a use rate of 84, 72, and 65%, respectively. Among the three groups surveyed, freshmen are more inclined to use WeChat, while seniors prefer QQ; their purpose to use new media differ greatly; more freshmen use new media for entertainment, while the seniors are interested in real-time news and daily communication over new media platforms. The QS results of “Time distribution of college students’ use of new media” show that over 50% of the respondents use new media for at least 5 h daily.

Thereupon, the following problems are summarized in the cultivation of college students’ entrepreneurial values and entrepreneurial ability under the new media environment:

(1) Teaching thinking and teaching philosophy are relatively backward.

Given the new media environment, the school is still mainly responsible for the cultivation of college students’ entrepreneurial values and entrepreneurial ability, yet family support is also essential. Currently, however, only a few school departments are involved in the organization and coordination of entrepreneurial courses, leading to an incomplete IEE system.

(2) In terms of the teaching content, few practices have been implemented compared with theories of IEE in higher institutions, which hinders the cultivation of students’ entrepreneurial values and entrepreneurial ability but also has a negative impact on their personal development.

### Discussion

This paper specifically explores the entrepreneurial values of students from a university in Shaanxi Province, makes an in-depth analysis from the aspects of the factors affecting college students’ entrepreneurial choices, the solutions to entrepreneurial risks, and the factors affecting entrepreneurial failure, and draws the corresponding conclusions and suggestions. Liu (2019) believed that IEE was the primary guarantee for the active innovation mechanism; the research mainly analyzed the factors affecting the entrepreneurship of students in Economics majors at this stage, thereby pointing out a new choice of employment for students majoring in Economics. Wang (2020a) required higher institutions to strengthen the cultivation of students’ innovative and entrepreneurial ability, as well as the practical operation ability and risk response-ability in entrepreneurship from the social reality. Peter (2020) pointed out that entrepreneurship could bring a new way of thinking through different organizational structures, but there were relatively few studies on the theoretically related variables, especially, empirical research. Therefore, studying the impact of entrepreneurial spirit on entrepreneurial enthusiasm was an attempt to break through the research limitations, variable research structure, and empirical research innovation. Tian (2019) believed that individuals with strong entrepreneurial enthusiasm were more willing to conduct effective entrepreneurial practice, through which their risk-bearing ability and challenging spirit in entrepreneurship would be improved. By comparing the results of this paper with the theories of the above literature, it can be found that each study has focused on different subjects. Thereupon, this paper defines the research object as college students through the theoretical research and review of related variables and then carries out data collection, analysis, and empirical research on this special group with great potentials. Based on data support and model calculation, this paper discusses the relationship and intermediary mechanism between entrepreneurial spirit and college students’ entrepreneurial enthusiasm, tries to make breakthroughs and prospects in new entrepreneurial perspectives, directions, and positions, transform entrepreneurial behavior for college students’ entrepreneurial enthusiasm, improve entrepreneurial negative attitude and entrepreneurial management ability, provide antecedents, theoretical basis, and practical suggestions for the success of entrepreneurship.

## Cultivating Strategies of College Students’ Innovation and Entrepreneurship Education Under the New Media

The above research suggests that IEE in Chinese higher institutions is in the theoretical teaching stage and lacks practical teaching reform to effectively cultivate students’ entrepreneurial concepts and entrepreneurial ability. In addition to strengthening the cultivation of college students’ entrepreneurial psychology and entrepreneurial spirit, the cultivation strategy might also include the following aspects.

(1) Students might benefit from unified ideas to better understand IEE. It is the responsibility and obligation of higher institutions to cultivate talents with an entrepreneurial spirit for the country through IEE. There is a need for higher institutions to help students understand the basic theory and business law in IEE and improve their environmental adaptability and risk controllability. Above all, it is essential to cultivate students’ entrepreneurial spirit through IEE, namely, enterprising spirit, professionalism, integrity, dedication, and sense of social responsibility.
(2) There is a need to formulate an innovation training plan and build an integral IEE system to promote IEE. To cultivate students with a more comprehensive entrepreneurial spirit, there should be a series of education of entrepreneurship management theory and entrepreneurship training. The teaching content should be customized for students in different grades.
(3) It might benefit the students to cultivate entrepreneurial awareness in the entrepreneurial atmosphere through various school activities, which provides students with reference and guidance for entrepreneurship, as well as a platform to exercise their entrepreneurial ability, thus stimulating students’ innovative awareness and entrepreneurial spirit in the form of community organizations.
(4) Entrepreneurial resources in higher institutions might be integrated to improve entrepreneurial management efficiency. Multiple factors can be combined with the cultivation of IEE in higher institutions, such as the support of national entrepreneurship policies, the specific guidance of enterprises, and various organizations, to form a complete and effective IEE system that integrates existing entrepreneurship resources and improves entrepreneurship management efficiency.

The optimization strategies (1)–(4) are favorable for the implementation of effective and efficient IEE and training under the new media era and have a positive impact on the cultivation of students’ entrepreneurial values and entrepreneurial ability.

## Conclusion

The purpose is to analyze the impact of entrepreneurial spirit on the cultivation of contemporary students’ entrepreneurial values and entrepreneurial ability under the background of new media, study the existing problems in the cultivation of IEE in the higher institution through QS. The QS results show in terms of the question “What are the factors affecting entrepreneurial values,” 39.43% of respondents choose the “Models of the successful entrepreneur,” showing that the successful entrepreneur model can encourage students to receive IEE; 28.94% of the students choose to “Achieve their own life goals”; those who have chosen “Government influence” or “School influence” both exceed 15%. The students’ answers to “The reasons for entrepreneurial failure” indicate that 40.37, 31.9, 25.98, and 11.75% of the respondents think they lack financial support, business skills, ability, or understanding of policies and laws, respectively; entrepreneurship is a process of practice, and IEE needs to be combined with practical activities; the skills and ability of entrepreneurs play an important role in the entrepreneurial process and have been recognized by students. Additionally, financial support affects the whole life cycle of entrepreneurship. This paper provides great help for the cultivation of entrepreneurial values and entrepreneurial ability of college students in the future. However, there are some limitations: at the theoretical level, the results of targeted suggestions and policies have not been verified and supported by other data, and there is no deeper theoretical and practical exploration. The proposed training strategies on students’ entrepreneurial values and entrepreneurial ability can provide a new theoretical basis and optimization direction for IEE in higher institutions and help students receive better IEE in the future.

## Data Availability Statement

The raw data supporting the conclusions of this article will be made available by the authors, without undue reservation.

## Ethics Statement

The studies involving human participants were reviewed and approved by South China Normal University Ethics Committee. The patients/participants provided their written informed consent to participate in this study. Written informed consent was obtained from the individual(s) for the publication of any potentially identifiable images or data included in this article.

## Author Contributions

Both authors listed have made a substantial, direct, and intellectual contribution to the work, and approved it for publication.

## Conflict of Interest

The authors declare that the research was conducted in the absence of any commercial or financial relationships that could be construed as a potential conflict of interest.

## Publisher’s Note

All claims expressed in this article are solely those of the authors and do not necessarily represent those of their affiliated organizations, or those of the publisher, the editors and the reviewers. Any product that may be evaluated in this article, or claim that may be made by its manufacturer, is not guaranteed or endorsed by the publisher.
